# The Evolution of Extended Platelet-Rich Fibrin Membranes for Socket Grafting: Part Two: A Randomized Clinical Trial Comparing These Membranes with Collagen Membranes

**DOI:** 10.3390/dj14010045

**Published:** 2026-01-12

**Authors:** Nathan E. Estrin, Paras Ahmad, Troy B. Tran, Alan Rene Espinoza, Ryan Holmes, Jean-Claude Imber, Nima Farshidfar, Richard J. Miron

**Affiliations:** 1 College of Dentistry and Dental Clinics, The University of Iowa, Iowa City, IA 52242, USA; drnathan@estrinperiodontics.com; 2 School of Dental Medicine, Lake Erie College of Osteopathic Medicine, Bradenton, FL 34211, USA; troybtran@gmail.com; 3 Department of Research, Advanced PRF Education, Jupiter, FL 33477, USA; paras@themironlab.com (P.A.); rick@themironlab.com (R.J.M.); 4 Private Practice, Global Dental Baja, Mexicali 21200, Mexico; dentistalanrene@gmail.com; 5 True View Dental Radiology PLLC, Boise, ID 83709, USA; ryan.holmes@trueviewdentalradiology.com; 6 Private Practice, Holmes Dental Radiology and Forensics PLLC, Boise, ID 83709, USA; 7 Department of Periodontology, University of Bern, 3010 Bern, Switzerland; jean-claude.imber@unibe.ch

**Keywords:** platelet-rich fibrin, albumin gel, albumin-PRF, bio-filler, extended platelet-rich fibrin

## Abstract

**Background:** Extended platelet-rich fibrin (e-PRF) membranes are a novel 100% autologous biomaterial with a longer resorption time (4–6 months) than traditional solid-PRF membranes (two weeks). In part 1 of this 2-part publication series, four clinical variations for using these novel e-PRF membranes for socket preservation were introduced. In this randomized clinical trial (RCT), all four iterations of e-PRF membranes were compared to traditional collagen membranes in alveolar ridge preservation for hard and soft tissue dimensional changes and early wound healing outcomes. **Methods:** A single-center RCT was conducted, including 55 patients requiring the extraction of a single tooth with planned implant placement. All sockets were grafted with a “sticky bone” (bone allograft mixed with PRF) and secured with either a collagen membrane (control) or e-PRF membranes utilizing the four variations present in Part 1 (both formed extra-orally or intra-orally, each with or without an overlying solid PRF membrane). The time of fabrication and application of each e-PRF iteration was recorded. Cone beam computed tomography was utilized to evaluate horizontal and vertical ridge dimensions at baseline and 3 months post-operatively, and soft tissue thickness was also measured at both time intervals utilizing an endodontic reamer. Early wound healing was recorded at 2 weeks, utilizing the Landry, Turnbull, and Howley Index by three blinded clinicians. **Results:** The results demonstrated that, at 3 months, the e-PRF membranes fabricated utilizing all 4 treatment variations demonstrated equal improvements in horizontal and vertical ridge dimensions and soft tissue thickness when compared to collagen membranes. Additionally, the membrane (*p* = 0.029) and membrane w/solid (*p* = 0.021) groups demonstrated statistically significant superior early wound healing compared to the collagen membrane group. Notably, the Bio-Filler groups demonstrated statistically significant reduction in fabrication/application time compared to the membrane groups. **Conclusions:** Within the limitations of this RCT, all e-PRF iterations performed comparably to collagen membranes in maintaining both hard and soft tissue ridge dimensions when combined with sticky bone, while also significantly improving soft tissue wound healing. Future RCTs with alternative grafting materials, direct wound-margin assessment, and evaluation of patient-reported outcomes are necessary to clarify the advantages of each membrane type.

## 1. Introduction

Successful dental implant therapy fundamentally depends on the careful management of post-extraction sites to minimize the inevitable dimensional changes that follow tooth extraction [1]. It is well established that nearly two-thirds of alveolar ridge resorption occur within the first six months post-extraction, with the most significant alterations observed during the early healing stage [2]. Without adequate ridge preservation measures, these volumetric reductions can undermine the optimal three-dimensional positioning and long-term stability of dental implants [3,4,5]. As a result, ridge preservation has emerged as a critical part of current implant therapy, typically involving the placement of bone graft particulates with barrier membranes to secure the graft material and prevent connective and epithelial tissue infiltration [6,7].

Platelet-rich Fibrin (PRF) has been widely incorporated into regenerative dental procedures due to its autologous nature and its ability to release concentrated growth factors that promote angiogenesis, enhance cellular migration, and expedite soft tissue healing [8,9,10,11]. Through modifying centrifugation protocols and collection tube technology, PRF can be prepared in both solid and liquid forms, hence expanding its clinical versatility [12,13]. Both preparations can be combined with bone grafts to fabricate “sticky bone”, offering superior handling features and supplementary biologic benefits [14,15,16]. Nonetheless, the utilization of solid PRF as a solo barrier membrane has been limited by its comparatively rapid degradation, usually within two weeks, making it unsuitable for applications mandating prolonged barrier functions [17]. Resultantly, traditional collagen or polytetrafluoroethylene (PTFE) membranes have remained the clinical standard [17].

In 2015, Kawase et al. [18] introduced a novel heat-compression technique to prolong the stability of PRF membranes beyond three weeks. This innovation was based on the hypothesis that controlled thermal denaturation of albumin within the PRF matrix could result in a slower degradation rate while maintaining biocompatibility [19]. The resulting albumin PRF (Alb-PRF) protocol aimed to preserve the inherent biological advantages of PRF while improving its longevity and mechanical integrity [20,21]. The evolution of this concept resulted in the formulation of extended PRF (e-PRF), which combines heat-denatured albumin produced through Bio-Heat technology with unheated injectable-PRF (i-PRF) from the concentrated buffy coat zone, hence incorporating both prolonged resorption features along with standard bioactivity found in PRF.

In a recent study, our group demonstrated clinical cases highlighting the use of e-PRF as an alternative to collagen membranes in ridge preservation, ridge augmentation, and for soft tissue coverage [20]. Furthermore, we have exhibited its effective application as an alternative to collagen membranes for coverage of the lateral window in maxillary sinus augmentation protocols [22]. However, the evidence supporting the use of e-PRF membranes remains limited and is largely confined to case series, highlighting the importance in conducting a well-designed randomized controlled clinical trial (RCT). Additionally, despite the proposed benefits, formulation of e-PRF presently requires around 30–40 min of preparation, comprising an 8 min centrifugation cycle, a 10 min denaturization time, cooling steps, followed by mixing of the albumin gel with liquid PRF, and an additional 15 min setting time. For addressing and overcoming this challenge, our recent study (Part I of this series) investigated the feasibility of intraoral membrane polymerization, hence decreasing chairside time [23]. This technique leverages the known clinical application of injectable e-PRF “Bio-Fillers” in other medical fields, such as in knee and temporomandibular joint injections, as well as facial esthetics, where fibrin polymerization is expedited at physiological temperature [20,24]. Considering that the transformation of fibrinogen to fibrin is a temperature-based, enzymatic mechanism, the intraoral environment may further accelerate membrane setting, potentially improving the clinical application of e-PRF and reducing chairside time.

In Part I of this series, we proposed that combining a solid PRF membrane exposed to the oral cavity with an underlying e-PRF membrane may provide supplementary advantages. Solid PRF membranes show superior mechanical robustness and handling properties compared to e-PRF and release their entire complement of autologous growth factors within two weeks before resorption. This aligns with the early stage of wound healing and may act to preserve the underlying e-PRF membrane, which remains unexposed and continues to serve as a secondary biologic barrier. Based on this, we hypothesized that a dual-layer configuration may improve graft stability and minimize particulate loss during socket preservation procedures.

Stemming from this concept, 4 characteristic variations in the e-PRF protocol emerged, fabricating the e-PRF either intraorally or extra-orally, each with or without an overlying solid PRF membrane. Even though these variations have garnered increasing clinical adoption and are being incorporated into postgraduate training and continuing education programs, no RCT has yet assessed their comparative efficacy. Hence, the present study was designed as the first RCT to directly compare four e-PRF variations within the traditional collagen membrane in alveolar ridge preservation. The primary outcome measure of this study was radiographic ridge width changes at 3 months post-extraction. Secondary outcomes included vertical ridge height changes, provider-assessed early wound healing, and membrane fabrication and application time.

## 2. Materials and Methods

### 2.1. Ethical Considerations and Study Design

Before the initiation of this study, ethical approval was obtained from the Sterling Institutional Review Board (IRB; Protocol ID: 13180-NEstrin) and registered on ClinicalTrial.gov (NCT:07281053) and reported in line with the CONSORT guideline [25]. This study was designed as a single-center, parallel group, RCT conducted in a private clinical setting (Lakewood Ranch Dental, Sarasota, FL, USA) from 20 February 2025 to 1 November 2025. A total of 70 participants were recruited requiring the extraction of a single tooth with subsequent implant placement were enrolled following informed consent. All participants underwent screening as per the predefined eligibility criteria. Exclusion criteria comprised active smoking, uncontrolled systemic disease (including diabetes with HbA1c > 7%), autoimmune or neurologic disorders, metabolic bone disease, pregnancy or lactation, and the use of systemic antibiotics within the past three months.

### 2.2. Study Groups

Participants were randomly assigned to one of five treatment groups utilizing a simple randomization procedure where group assignments were prepared in advance and drawn from a container at time of enrollment by a member of the research time with the allocation concealed from the operating clinician (N.E.) until time of membrane fabrication. Each group represented a unique socket preservation protocol: Group I (Control): Resorbable collagen membrane (standard of care); Group II: e-PRF used as a sole barrier membrane; Group III: Dual-layer configuration containing an e-PRF membrane overlaid with a solid PRF membrane; Group IV: e-PRF fabricated intraorally in gel form (Bio-Filler technique); and Group V: e-PRF fabricated intraorally and covered with a solid PRF membrane.

### 2.3. Surgical Protocol

Under local anesthesia, atraumatic tooth extraction was performed in all cases, with meticulous care to preserve the integrity of the surrounding alveolar bone and without reflection of mucoperiosteal flaps. After extraction, each socket was thoroughly degranulated using surgical curettes and/or carbide burs at low speed until a hard, bleeding osseous surface was achieved, ensuring complete removal of granulation tissue.

As a standard practice and due to the superior mechanical handling and wound healing ability [26], all socket grafting was carried out using sticky bone formulated by combining allograft particulate (FDBA, MinerOss, BioHorizons, Birmingham, AL, USA) with solid and liquid PRF as previously described [22,23]. The grafted sockets were then covered with either a resorbable collagen membrane (Mem-Lok^®^ Resorbable Collagen Membrane, BioHorizons, Birmingham, AL, USA) or one of the assigned e-PRF configurations prepared using the Bio-Heat protocol, as described in Part 1 of this series [23].

All membranes were stabilized using 3–0 chromic gut sutures, and primary closure was intentionally avoided to permit secondary epithelialization. Post-operatively, all patients were prescribed amoxicillin 500 mg three times daily for 7 days and ibuprofen 800 mg three times daily as needed for pain management. Moreover, each participant received a homeopathic oral care recovery kit (StellaLife^®^, StellaLife Inc., Chicago, IL, USA) to facilitate post-surgical healing [27]. Patients were recalled at 2 weeks for postoperative assessment and again at 3 months for clinical and radiographic evaluation before implant placement as consistent with previous studies [28,29]. All surgical procedures were performed by the same clinician (N.E.), a board-certified periodontist.

### 2.4. e-PRF Iteration Fabrication and Application Time

For all patients in the treatment groups, the time to fabricate and apply the e-PRF iteration was recorded. This included centrifugation, using the Bio-Heat to denature albumin, cooling the tubes, mixing the e-PRF with liquid-PRF, allowing the membrane to set, harvesting and flattening the solid-PRF membranes, and intraoral application of the membranes (Table S1). All procedures were timed by the same clinician performing the surgeries (N.E.) starting at the time of centrifugation and ending after completion of membrane application.

### 2.5. Radiographic Evaluation

Cone Beam Computed Tomography (CBCT, Ez3D-i, Vatech Green, Seoul, Republic of Korea) scans were acquired for all participants immediately after socket grafting and again at three months post-operatively. All scans were acquired utilizing standardized parameters and were assessed by an independent radiology service (Holmes Dental Radiology and Forensic, PLLC, Boise, ID, USA) and the radiographic measurements were completed by the same blinded oral and maxillofacial radiologist (R.H.) The baseline and follow-up datasets were spatially matched and registered within a common coordinate system using an open-source imaging software (3D Slicer, version 5.8.1; www.slicer.org) to ensure measuring consistency.

**Figure 1:**
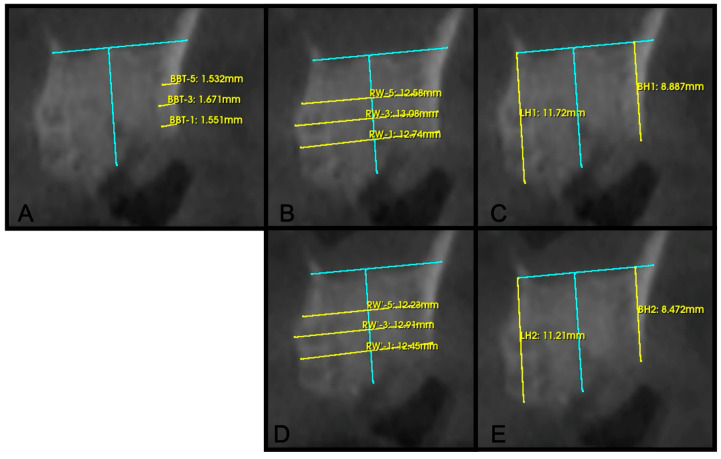
An example case of ridge preservation at site #15, demonstrating radiographic measurements. Immediate post-operative CBCT scan with: (**A**) Baseline buccal bone thickness at 1 mm (BBT-1), 3 mm (BBT-3), and 5 mm (BBT-5) apical to the crest; (**B**) Baseline measurements of ridge width at 1 mm (RW-1), 3 mm (RW-3), and 5 mm (RW-5) apical to the buccal crest; (**C**) Baseline measurements of Buccal height (BH) and Lingual Height (LH); (**D**) 3-month post-operative ridge width measurements at all levels; and (**E**) 3-month post-operative buccal and lingual height measurements. All measurements were taken with the same vertical and horizontal reference lines (in blue) at both time intervals.

Horizontal ridge width was measured on cross-sectional images at three standardized apical levels relative to the alveolar crest: 1 mm (RW-1), 3 mm (RW-3), and 5 mm (RW-5). Measurements were conducted perpendicular to a vertical reference line drawn through the midpoint of the grafted socket. Buccal height (BH) and lingual height (LH) were measured at both time points as the linear distance from the most apical portion of the socket to the most coronal portion of the alveolar ridge, parallel to the same vertical reference line. Baseline buccal bone thickness (BBT) was measured on cross-sectional images at 1 mm (BBT-1), 3 mm (BBT-3), and 5 mm (BBT-5) from the crest, parallel to a horizontal reference line (Figure 1).

All radiographic measurements were independently conducted and validated by calibrated investigators at Holmes Dental Radiology and Forensics, PLLC, to minimize measurement bias and ensure reproducibility.

### 2.6. Soft Tissue Thickness

Soft tissue thickness was measured utilizing a standardized mechanical probing procedure with a #40 endodontic reamer equipped with an adjustable rubber stopper. Measurements were taken at the buccal crest and 5 mm apical to the crest, both pre-operatively and at three months post-operatively, corresponding to the time of implant placement. Moreover, mid-occlusal soft tissue thickness (OSTT) was assessed at the three-month follow-up.

At each measurement point, the reamer was gently advanced perpendicularly through the mucosa until hard tissue contact was obtained. The distance from the tip of the reamer to the rubber stopper was then calculated utilizing a digital caliper (accuracy ±0.01 mm) to identify the soft tissue thickness. All measurements were carried out by a calibrated investigator under consistent positioning and lighting conditions to minimize variability.

### 2.7. Provider-Reported Healing Outcomes

At the two-week post-operative visit, intraoral photographs of each extraction site were taken using a digital camera, (Canon EOS R6, Ota, Tokyo, Japan). The images were independently assessed by three blinded co-authors (A.R.E., T.B.T., and N.F.) utilizing the Landry, Turnbull, and Howley Wound Healing Index (LWHI) [30]. Healing was scored on a scale from 1 to 5, with 1 indicating very poor healing and 5 indicating excellent healing. Inter-rater agreement statistics were not performed, and no formal consensus or adjudication process was conducted; rather, the mean of the three independent ratings was utilized for analysis.

### 2.8. Sample Size Calculation and Statistical Analysis

The original sample size was based on a ridge-width difference of 2.3 mm derived from Thalmair et al. [31], which compared ungrafted extraction sockets to membrane-protected sites. Nonetheless, since all study groups in the current study received sticky bone grafting and a barrier membrane, the expected inter-group difference was substantially smaller. Hence, this study should be interpreted as a non-inferiority trial assessing whether e-PRF membranes provide ridge preservation that is comparable to that of collagen membranes. Based on contemporary CBCT-based ridge preservation studies, differences of less than 0.5 mm are considered clinically negligible for dental implant placement and prosthetic emergence profile. A non-inferiority margin (Δ) of 0.5 mm was thereby chosen for HRW. Employing the observed pooled standard deviation (SD) of nearly 0.6 mm for ridge-width change, a sample size of 11 patients/group provides >80% power (α = 0.05) to exclude differences greater than 0.5 mm. The achieved enrollment of 11 patients/group was hence sufficient to exhibit the non-inferiority of e-PRF membranes relative to collagen.

### 2.9. Statistical Analysis

All statistical analyses were conducted using GraphPad Prism version 10.0 (GraphPad Software, San Diego, CA, USA). Data were first evaluated for normality utilizing the Shapiro–Wilk test. One-way analysis of variance (ANOVA) was used to identify overall differences among the research groups. When ANOVA showed statistical significance, appropriate post hoc tests were performed. Tukey’s multiple comparison test was used to compare all possible pairs of group means in this study, where no single control group was defined (i.e., membranes’ fabrication and application time). In contrast, Dunnett’s post hoc test was used in the analysis involving a defined control group (i.e., collagen) to specifically compare test groups with the control group while controlling for type I error across multiple comparisons. Results were expressed as mean ± standard deviation (SD) with lower to upper limit of 95% confidence interval (95% CI), and statistical significance was set at *p* < 0.05.

#### Multiplicity and Control of Type I Error

For addressing multiplicity, the statistical analysis was structured hierarchically. The HRW change (i.e., primary endpoint) was considered confirmatory and controlled at a two-sided α = 0.05 utilizing Dunnett’s test vs. collagen. All secondary outcomes, such as membrane fabrication time, wound healing index, soft tissue thickness, and vertical ridge height, were predefined as exploratory and intended for hypothesis formation. For these endpoints, multiplicity adjustments were used within individual outcome families utilizing Tukey’s post hoc or Dunnett’s analyses; however, no global family-wise error correction across all secondary outcomes was employed, consistent with exploratory trial design. Hence, *p*-values for secondary outcomes should be interpreted descriptively rather than as definitive evidence of superiority.

## 3. Results

### 3.1. Primary Characteristics of Study Participants

A total of 55 patients (27 females and 28 males) with a mean age of 67.3 ± 10.5 years (range: 42–82 years) were included. Participants were treated at several tooth sites, with the majority involving molars (*n* = 39) and the remainder premolars (*n* = 16). Distribution across test groups was comparatively balanced, with 11 patients per research group (Table S2).

### 3.2. Horizontal Ridge Width Outcomes

Across all measured heights, ridge reduction was usually comparable between the control (collagen) and test groups. At ΔRW-1, control (1.31 ± 0.74 mm) and the membrane (1.29 ± 0.88 mm; 95% CI: −0.677 to 0.717) demonstrated slightly higher reductions than membrane w/solid (0.99 ± 0.41 mm; −0.375 to 1.019). In comparison, Bio-Filler (1.40 ± 0.66 mm; −0.787 to 0.607) was marginally higher than the control, and Bio-Filler w/solid (1.27 ± 0.42 mm; −0.655 to 0.739) was similar to the control group (Table 1).

At ΔRW-3, all groups exhibited lower reductions than at ΔRW-1, with control (0.89 ± 0.58 mm) and Bio-Filler (0.90 ± 0.57 mm; −0.653 to 0.627) slightly higher than membrane w/solid (0.77 ± 0.57 mm; −0.544 to 0.736), and membrane (0.79 ± 0.66 mm; −0.544 to 0.736) and Bio-Filler w/solid (0.88 ± 0.59 mm; −0.630 to 0.650) were comparable to the control group (Table 1).

At ΔRW-5, Bio-Filler (0.30 ± 0.30 mm; −0.207 to 1.034) exhibited the least reduction, membrane w/solid (0.74 ± 0.74 mm; −0.391 to 0.849) and Bio-Filler w/solid (0.60 ± 0.60 mm; −0.476 to 0.765) were intermediate, and control (0.94 ± 0.49 mm) and membrane (0.69 ± 0.69 mm; −0.415 to 0.826) showed slightly higher reductions. Overall, differences were small, and none of the groups differed significantly from the control group (all *p* > 0.05) (Table 1).

### 3.3. Buccal and Lingual Height Outcomes

For BH, mean values were slightly higher in Bio-Filler (1.20 ± 0.64 mm; −0.720 to 0.674) and Bio-Filler w/solid (1.19 ± 0.93 mm; −0.710 to 0.683) compared to control (1.17 ± 0.53 mm) and membrane (1.15 ± 0.53 mm; −0.676 to 0.718), with membrane w/solid exhibiting slightly lower height (0.81 ± 0.51 mm; −0.336 to 1.058) (Table 2).

For LH, Bio-Filler (0.77 ± 0.75 mm; −0.668 to 0.541) and Bio-Filler w/solid (0.71 ± 0.71 mm; −0.626 to 0.583) showed the highest values, control (0.70 ± 0.37 mm) and membrane (0.46 ± 0.46 mm; −0.646 to 0.564) were intermediate, and membrane w/solid (0.40 ± 0.40 mm; −0.335 to 0.875) was the lowest. No statistically significant differences were found between the control and test groups (*p* > 0.05) (Table 2).

### 3.4. Soft Tissue Outcomes

**Figure 2:**
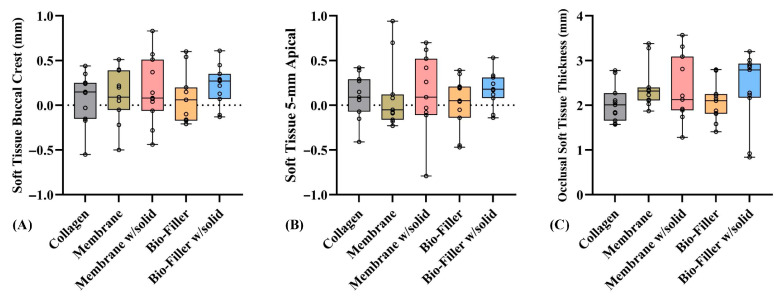
Soft tissue-associated outcomes: (**A**) soft tissue buccal crest; (**B**) soft tissue 5 mm apical; and (**C**) occlusal soft tissue thickness.

Mean soft tissue buccal crest changes were usually comparable across all groups. The control group demonstrated a mean change of 0.08 ± 0.29 mm, slightly lower than the membrane (0.11 ± 0.29 mm; −0.342 to 0.295) and similar to Bio-Filler (0.07 ± 0.28 mm; −0.308 to 0.329) and Bio-Filler w/solid (0.22 ± 0.22 mm; −0.456 to 0.181), while membrane w/solid showed a slightly higher change (0.17 ± 0.38 mm; −0.401 to 0.236). None of the differences between the control and test groups were statistically significant (all *p* > 0.05; MDs: −0.137 to 0.011 mm) (Figure 2A).

Mean soft tissue changes at 5 mm apical to the crest were small and comparable across all groups. The control group demonstrated a mean change of 0.10 ± 0.25 mm, slightly lower than the membrane (0.09 ± 0.38 mm; −0.334 to 0.353) and similar to Bio-Filler (0.03 ± 0.29 mm; −0.273 to 0.414) and Bio-Filler w/solid (0.18 ± 0.19 mm; −0.421 to 0.266), while Membrane w/solid (0.14 ± 0.43 mm; −0.379 to 0.308) had the greatest mean change. None of these differences were statistically significant (all *p* > 0.05; MDs: −0.077 to 0.071 mm) (Figure 2B).

Mean OSTT was highest in the membrane w/solid group (2.35 ± 0.73 mm; −0.957 to 0.333), followed by the membrane (2.40 ± 0.49 mm; −1.007 to 0.283), Bio-Filler w/solid (2.37 ± 0.81 mm; −0.978 to 0.311), Bio-Filler (2.07 ± 0.44 mm; −0.677 to 0.613), and the control group (2.04 ± 0.42 mm). Although the membrane and membrane w/solid demonstrated slightly greater thickness compared to the control group, no statistically significant differences were observed between the control and test groups (all *p* > 0.05; MDs: −0.362 to −0.032 mm) (Figure 2C).

### 3.5. Wound Healing Outcomes

**Figure 3:**
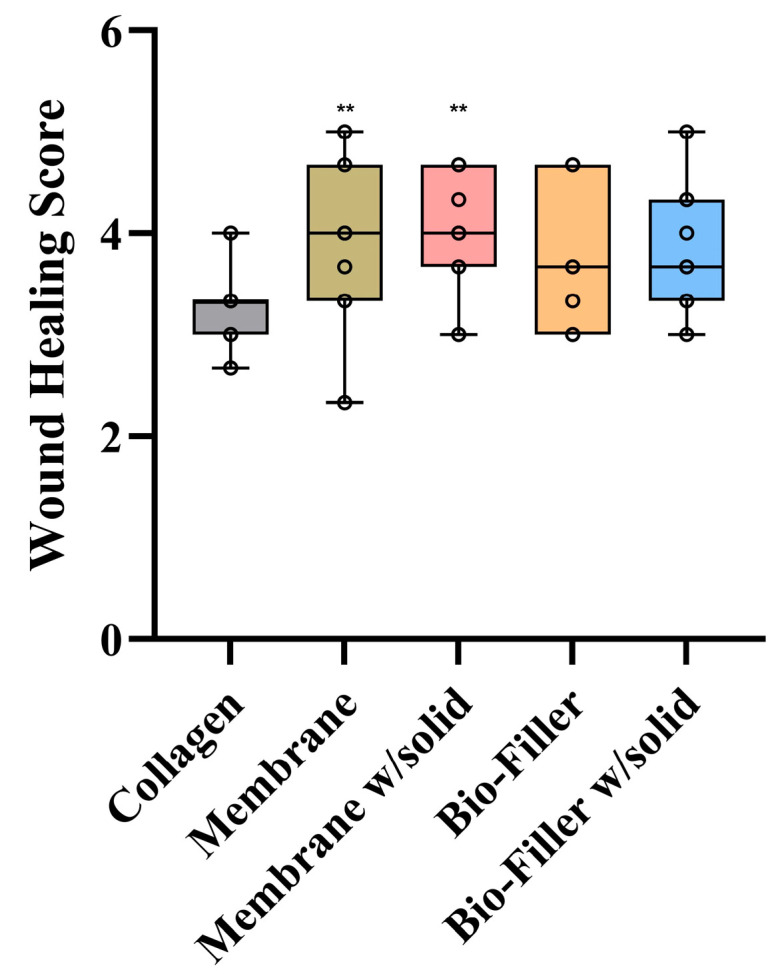
Wound healing scores. ** Indicates statistically significant difference when compared with the collagen (control) group (*p* < 0.05).

Mean wound healing scores were highest in the membrane w/solid group (3.97 ± 0.60; −1.430 to −0.089), followed by the membrane (3.94 ± 0.79; −1.398 to −0.057), Bio-Filler w/solid (3.88 ± 0.58; −1.337 to 0.004), Bio-Filler (3.70 ± 0.67; −1.157 to 0.184), and lowest in the control group (3.21 ± 0.40). While the membrane w/solid and e-PRF membranes exhibited slightly better wound healing than the control group, all test groups were comparable overall. Statistically significant differences were observed between the control and the membrane (*p* = 0.029) as well as membrane w/solid (*p* = 0.021) (Figure 3); however, these findings should be interpreted cautiously since no inter-rater reliability was conducted.

### 3.6. Membrane Fabrication and Application Outcomes

**Figure 4:**
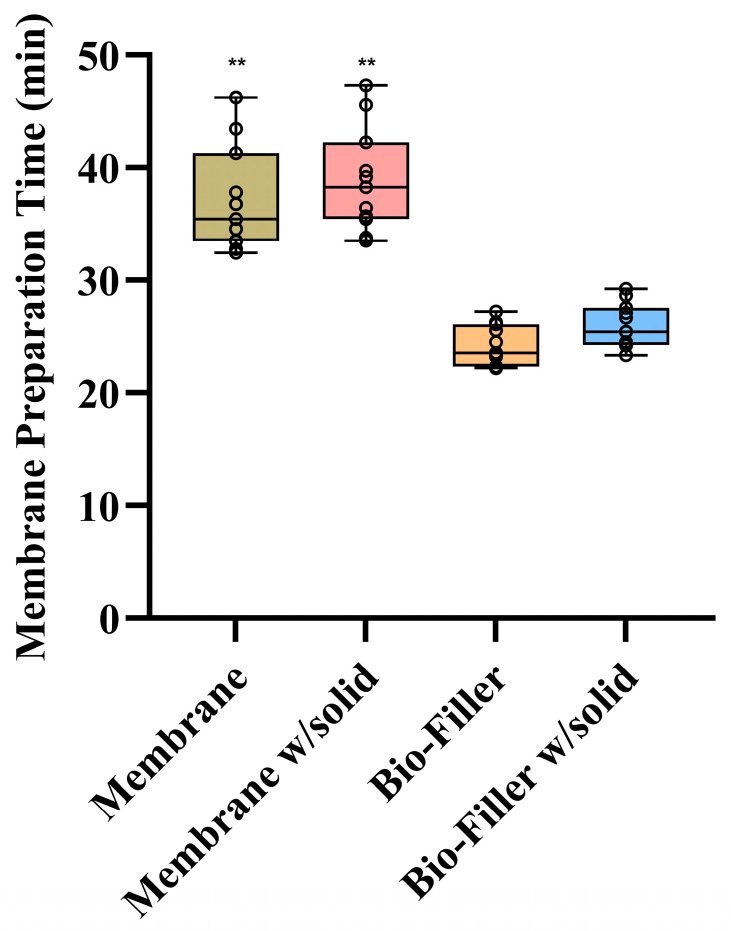
Fabrication and application outcomes of different e-PRF configurations used in this study. ** Shows statistically significant difference between the other two test groups of *p* < 0.0001.

Statistically significant inter-group differences were found in membrane fabrication and application times (*p* < 0.0001) (Figure 4, Table S3). Pairwise comparisons demonstrated that both the Bio-Filler (24.43 ± 1.77 min and Bio-Filler w/solid (26.20 ± 0.63 min needed significantly less time than the membrane (37.53 ± 4.63 min) and membrane w/solid (39.35 ± 4.48 min) groups (all *p* < 0.0001). However, no statistically significant differences were found between the two membrane-based (*p* > 0.05) or between the Bio-Filler preparations (*p* > 0.0001). Overall, both the membrane-based preparations required significantly longer formulation times than the Bio-Filler formulations (membrane vs. Bio-Filler: *t* = 8.35 min; membrane vs. Bio-Filler w/solid: *t* = 7.11 min; membrane w/solid vs. Bio-Filler *t* = 9.80 min; membrane w/solid vs. Bio-Filler w/solid: *t* = 8.50; all *p* < 0.0001).

## 4. Discussion

This RCT is the first of its kind to directly compare four e-PRF membrane configurations with a traditional collagen membrane for application in alveolar ridge preservation. The primary outcomes demonstrated no statistically significant differences in radiographic ridge dimensions or soft tissue thickness among the control and test groups at three months. Notably, no statistically significant differences were found when comparing all four e-PRF iterations to the collagen membrane regarding horizontal ridge width, buccal and lingual height, and soft tissue thickness outcomes. Nevertheless, clinician-reported wound healing evaluations favored the e-PRF configurations over collagen, indicating a possible biological benefit of these configurations in early wound healing. One significant limitation of this study is that all radiographic measurements were performed by a single oral and maxillofacial radiologist without duplicate or triplicate measurements. Future studies should incorporate multiple calibrated examiners to assess intra-rater reliability and perform duplicate or triplicate measurements to verify reproducibility and improve the validity of the radiographic outcomes.

The lack of significant differences in ridge dimensional stability across groups is likely attributable, at least in part, to the universal use of “sticky bone” in all test groups. The combination of particulate allograft with solid and liquid PRF is considered to improve graft cohesion, decrease particulate migration, and enhance osteoconductive stability [32,33,34,35]. These properties may have attenuated the effect of membrane type on ridge preservation outcomes and obscured potential differences between collagen and e-PRF membranes. As a standard practice, “sticky bone” was utilized due to its superior mechanical handling and wound healing ability [26]. However, it represents a methodological limitation when assessing barrier membrane performance, as it decreases the dependency on membrane integrity to retain graft material. Another limitation of the current study is the short follow-up time of only 3 months. While this time period is clinically relevant for implant planning and consistent with previous studies [28,29], additional dimensional changes may continue to occur up to 12 months following an extraction [3]. Future clinical trials excluding “sticky bone” or including study arms without a graft cohesion strategy with a longer-term follow-up are needed for a more definitive comparison between the barrier function of collagen and e-PRF membranes in ridge preservation. Additionally, comparison of e-PRF membranes to other common membranes such as PTFE and with histological analysis may further elucidate the clinical advantages of these autologous barriers for ridge preservation.

Regarding soft tissue healing, all e-PRF groups were associated with statistically higher LWHI wound healing scores when compared to the control group. Among the e-PRF configurations, the extra-orally formulated e-PRF membrane and membrane w/solid achieved the highest scores, indicating that ex vivo, controlled polymerization may improve membrane stability and early wound healing. Conversely, the intraorally prepared “Bio-Filler” or “injectable e-PRF” approaches rely on a partially uncontrolled polymerization environment, susceptible to disruption by salivary flow, mastication, or speech [23]. Therefore, increased patient compliance may be necessary for patients to avoid disrupting the polymerization of these membranes allowing them to set intra-orally. These clinical variables may account for the slightly higher variability found in wound healing outcomes among the Bio-Filler groups. Future clinical studies are necessary to further evaluate the mechanisms occurring during intra-oral polymerization such as if the patient’s saliva or oral pH has any effect on polymerization. Moreover, groups utilizing a solid PRF overlay showed decreased variance and slightly enhanced performance, suggesting the biological and structural advantages of a dual-layer membrane technique. Furthermore, solid PRF confers early release of growth factors while offering retention and mechanical protection of the underlying e-PRF membranes as it continues to mature.

An important aspect affecting the clinical applicability of these protocols is the difference in chairside time required for membrane fabrication and application. While the membrane-based formulations showed superior wound healing outcomes in this study, the extended extra-oral preparation time may limit their practicality in fast-paced clinical settings. Contrarily, the injectable, intra-orally prepared configurations significantly reduced chairside time by avoiding the 10–15 min setting period required for membrane polymerization. Additional limitations of solid-PRF encompass the need for multiple collection tubes and the added manipulation time for flattening and layering. Although the injectable e-PRF approach depends more on patient compliance during in situ setting, its procedural efficiency and simplicity provide significant clinical advantages. Future studies should assess whether the reduction in fabrication time impacts long-term regenerative outcomes or membrane stability.

Despite the observed benefits in wound healing, it is imperative to note that only the LWHI was utilized to assess soft tissue healing, and no direct measurements of keratinized tissue migration or wound margin closure were conducted. This is an important limitation of this study, as previous reports have demonstrated that despite expediting early epithelial maturation, traditional PRF membranes do not always enhance wound closure rates in comparison with collagen-based dressings or spontaneous healing [36,37,38]. Nonetheless, the significantly prolonged resorption duration of e-PRF membranes (4 to 6 months) in comparison to solid PRF (7 to 10 days) raises the possibility that prolonged biological activity may result in differences in mucosal sealing over time, a hypothesis that requires future investigation. Additionally, given the fact that i-PRF can be utilized as a vehicle for drug delivery [39], a similar concept may theoretically apply to e-PRF membranes as biomolecules of the clinician’s choice can be mixed in with the albumin gel and the liquid-PRF prior to polymerization. This provides an exciting avenue for future research as e-PRF membranes have the potential to be enriched with selected biomolecules such as antibiotics [40], vitamins [41], and/or extracellular vesicles [42]. However, controlled clinical studies are required to evaluate the clinical efficacy of enriching e-PRF and utilizing the membranes for drug entrapment and delivery.

Beyond their functionality as physical barriers, e-PRF membranes provide a biologically active alternative to inert collagen membranes [20,22,23]. Contrary to collagen, which mainly acts as a structural scaffold, e-PRF membranes release sustained levels of growth factors, including transforming growth factor-beta (TGF-β), vascular endothelial growth factor (VEGF), and platelet-derived growth factor (PDGF), over a prolonged duration, enhancing angiogenesis, fibroblast proliferation, and extracellular matrix (ECM) remodeling, all of which may promote early mucosal coverage and expedite soft tissue healing [10,43,44]. This may alleviate the predisposition of post-surgical infection, graft exposure, and patient morbidity, all crucial variables of patient satisfaction and clinical success.

Importantly, across all soft tissue endpoints, the observed mean differences versus collagen were extremely small, ranging between −0.36 and 0.07, with all 95% Cis overlapping zero (Figure 2). Hence, all differences and their 95% CIs fell well below 0.3 mm, which is widely considered the threshold below which alterations in mucosal thickness do not affect peri-implant stability, prosthetic contouring, or emergence profile. Therefore, this small difference is unlikely to be clinically significant, even when measured using a high-precision mechanical probe.

Overall, these outcomes indicate that e-PRF membranes represent a biologically active, clinically viable, and fully autologous alternative to conventional collagen membranes in socket preservation. Even though soft tissue thickness and radiographic results were comparable across groups, the superior early wound repair found with e-PRF merits further investigation. Prospective clinical studies should assess long-term implant survival, patient-reported outcomes, and direct measurement of soft tissue wound closure. Furthermore, the incorporation of histological analysis and non-sticky bone graft cohorts would yield clarity related to the effect of membrane selection on both soft and hard tissue healing. Moreover, since multiple secondary outcomes were assessed, soft tissue and wound healing outcomes should be interpreted as hypothesis-generating rather than confirmatory. Another limitation is associated with the wound healing evaluation. While three blinded assessors independently scored all sites utilizing the Landry-Turnbull-Howley index, inter-rater agreement statistics were not conducted, and no formal adjudication or consensus analysis was utilized. Since wound healing was the only outcome demonstrating statistically significant between-group differences, this lack of reliability evaluations limits the strength of inference and highlights that these outcomes should be considered exploratory rather than definitive.

## 5. Conclusions

Within the limitations of this study, no statistically significant differences were found when comparing all four e-PRF membranes to traditional collagen membranes in maintaining ridge dimensions and soft tissue thickness when used with sticky bone. Importantly, e-PRF membranes demonstrated superior early wound healing, supporting their role as a biologically active, fully autologous alternative to conventional barriers. Future RCTs incorporating direct wound margin evaluations, patient-reported outcomes, and methods without sticky bone are warranted to validate these outcomes and optimize the application of e-PRF, especially in conjunction with solid PRF and controlled extra-oral preparation, for improved clinical results in socket preservation.

## Figures and Tables

**Table 1:** Horizontal ridge width reduction changes at 3 months post-extraction and initial buccal bone thickness expressed as Mean ± Standard Deviation (Lower to upper limit of 95% CI).

**Δ Horizontal Ridge Width**			
**Groups**	**1 mm**	**3 mm**	**5 mm**
Collagen	1.31 ± 0.74 (Reference)	0.89 ± 0.58 (Reference)	0.94 ± 0.49 (Reference)
Membrane	1.29 ± 0.88 (−0.677 to 0.717)	0.79 ± 0.66 (−0.544 to 0.736)	0.78 ± 0.69 (−0.415 to 0.826)
Membrane w/solid	0.99 ± 0.41 (−0.375 to 1.019)	0.77 ± 0.57 (−0.522 to 0.758)	0.71 ± 0.74 (−0.391 to 0.849)
Bio-Filler	1.40 ± 0.66 (−0.787 to 0.607)	0.90 ± 0.57 (−0.653 to 0.627)	0.53 ± 0.30 (−0.207 to 1.034)
Bio-Filler w/solid	1.27 ± 0.42 (−0.655 to 0.739)	0.88 ± 0.59 (−0.630 to 0.650)	0.80 ± 0.60 (−0.476 to 0.765)
**Buccal Bone Thickness**			
**Groups**	**1 mm**	**3 mm**	**5 mm**
Collagen	0.90 ± 0.67 (Reference)	1.00 ± 0.63 (Reference)	1.12 ± 0.67 (Reference)
Membrane	1.01 ± 0.68 (0.55 to 1.47)	1.17 ± 0.66 (0.73 to 1.61)	1.29 ± 0.68 (0.83 to 1.75)
Membrane w/solid	0.92 ± 0.64 (0.49 to 1.35)	1.08 ± 0.68 (0.62 to 1.54)	1.23 ± 0.73 (0.74 to 1.72)
Bio-Filler	0.92 ± 0.85 (0.35 to 1.49)	1.07 ± 0.94 (0.44 to 1.70)	1.21 ± 0.98 (0.55 to 1.87)
Bio-Filler w/solid	0.96 ± 0.56 (0.59 to 1.33)	1.08 ± 0.55 (0.71 to 1.45)	1.23 ± 0.63 (0.81 to 1.65)

One-way ANOVA with Dunnett’s post hoc test revealed no statistically significant differences between the control and test groups, nor among the test groups themselves (*p* > 0.05).

**Table 2:** Buccal and lingual height changes at 3 months post-extraction expressed as Mean ± Standard Deviation (Lower to upper limit of 95% CI).

Groups	Buccal Height (mm)	Lingual Height (mm)
Collagen	1.17 ± 0.53 (Reference)	0.70 ± 0.37 (Reference)
Membrane	1.15 ± 0.53 (−0.676 to 0.718)	0.75 ± 0.46 (−0.646 to 0.564)
Membrane w/solid	0.81 ± 0.51 (−0.336 to 1.058)	0.43 ± 0.40 (−0.335 to 0.875)
Bio-Filler	1.20 ± 0.64 (−0.720 to 0.674)	0.77 ± 0.75 (−0.668 to 0.541)
Bio-Filler w/solid	1.19 ± 0.93 (−0.710 to 0.683)	0.73 ± 0.71 (−0.626 to 0.583)

One-way ANOVA with Dunnett’s post hoc test revealed no statistically significant differences between the control and test groups, nor among the test groups themselves (*p* > 0.05).

## Data Availability

Data supporting the findings of this study are available from the corresponding author upon reasonable request.

## Supplementary Materials

The following supporting information can be downloaded at: https://www.mdpi.com/article/10.3390/dj14010045/s1, Table S1: Outline of fabrication steps for each e-PRF membrane iteration. Table S2: Primary features of the study participants. Table S3: Time of fabrication and application of membrane type.

## Abbreviations

The following abbreviations are used in this manuscript:

PRF	Platelet-rich fibrin
PTEE	Polytetrafluoroethylene
alb-PRF	Albumin platelet-rich fibrin
e-PRF	Extended platelet-rich fibrin
RCT	Randomized clinical trial
IRB	Institutional Review Board
CBCT	Cone beam computed tomography
RW	Ridge width
BH	Buccal height
LH	Lingual height
BBT	Buccal bone thickness
OSTT	Occlusal soft tissue thickness
LWHI	Landry, Turnbull, and Howley Wound Healing Index
HRW	Horizontal ridge width
SD	Standard deviation
MDs	Mean differences
TGF-β	Transforming growth factor-Beta
VEGF	Vascular endothelial growth factor
PDGF	Platelet-derived growth factor
