# Supplementary material for: The Evolution of Extended Platelet-Rich Fibrin Membranes for Socket Grafting: Part Two: A Randomized Clinical Trial Comparing These Membranes with Collagen Membranes
Source: Dent J (Basel). 2026 Jan 12;14(1):45. doi: 10.3390/dj14010045 (PMC12840002; doi:10.3390/dj14010045)
Supplement: Supplementary file 1 [file dentistry-14-00045-s001.zip › dentistry-4006568-supplementary.pdf]

Supplementary Table

Table S1. Outline of fabrication steps for each e-PRF membrane iteration.

Technique	Centrifugation	Tubes	BioHeat (75 °C)/BioCool(8 °C)	Extraoral setting	Dual Layer w/Solid-PRF	Suturing
Membrane	700g for 8 min	2 Blue tubes	10 min/2min	Yes (~15 min)	No	After application
Membrane w/solid	700g for 8 min	2 Blue tubes, 1 red	10 min/2min	Yes (~15 min)	Yes	After application
Bio-Filler	700g for 8 min	2 Blue tubes	10 min/2min	No	No	Before application
Bio-Filler w/solid	700g for 8 min	2 Blue tubes, 1 red	10 min/2min	No	Yes	After application

Table S2. Primary features of the study participants.

Patient	Gender	Age	Tooth #	Tooth Type	Membrane
1	F	79	#3	Molar	ePRF Membrane
2	M	82	#15	Molar	ePRF Membrane
3	F	75	#4	Premolar	ePRF Membrane
4	F	78	#19	Molar	ePRF Membrane
5	M	56	#31	Molar	ePRF Membrane
6	M	47	#13	Premolar	ePRF Membrane
7	M	77	#19	Molar	ePRF Membrane
8	F	69	#30	Molar	ePRF Membrane
9	M	78	#3	Molar	ePRF Membrane
10	M	61	#14	Molar	ePRF Membrane
11	M	61	#18	Molar	ePRF Membrane
12	M	53	#18	Molar	Solid Overtop
13	M	78	#2	Molar	Solid Overtop
14	M	79	#15	Molar	Solid Overtop
15	M	69	#15	Molar	Solid Overtop
16	M	61	#14	Molar	Solid Overtop
17	M	61	#31	Molar	Solid Overtop
18	F	56	#4	Premolar	Solid Overtop
19	M	70	#3	Molar	Solid Overtop
20	M	70	#4	Premolar	Solid Overtop
21	M	58	#30	Molar	Solid Overtop
22	F	77	#18	Molar	Solid Overtop
23	F	68	#3	Molar	Biofiller
24	M	58	#19	Molar	Biofiller
25	M	75	#14	Molar	Biofiller
26	F	58	#18	Molar	Biofiller
27	F	60	#3	Molar	Biofiller
28	F	60	#30	Molar	Biofiller
29	F	54	#30	Molar	Biofiller
30	M	57	#3	Molar	Biofiller
31	F	50	#30	Molar	Biofiller
32	M	66	#29	Premolar	Biofiller

Supplementary Table

33	F	42	#3	Molar	Biofiller
34	M	55	#3	Molar	BioFiller w/ Solid
35	M	68	#13	Premolar	BioFiller w/ Solid
36	M	74	#20	Premolar	BioFiller w/ Solid
37	M	75	#18	Molar	BioFiller w/ Solid
38	F	73	#2	Molar	BioFiller w/ Solid
39	F	82	#19	Molar	BioFiller w/ Solid
40	F	82	#20	Premolar	BioFiller w/ Solid
41	M	80	#5	Premolar	BioFiller w/ Solid
42	M	79	#4	Premolar	BioFiller w/ Solid
43	F	60	#2	Molar	BioFiller w/ Solid
44	F	74	#30	Molar	BioFiller w/ Solid
45	M	73	#3	Molar	Collagen
46	F	62	#4	Premolar	Collagen
47	M	68	#18	Molar	Collagen
48	M	68	#19	Molar	Collagen
49	F	71	#2	Molar	Collagen
50	F	71	#3	Molar	Collagen
51	F	71	#5	Premolar	Collagen
52	F	58	#13	Premolar	Collagen
53	M	77	#3	Molar	Collagen
54	F	57	#3	Molar	Collagen
55	F	75	#3	Molar	Collagen

Tables S3. Time of fabrication and application of membrane type.

Treatment Group	Average Time (minutes)
e-PRF Membrane	37.06 ± 4.65
Membrane w/solid	38.83 ± 4.59
Bio-Filler	24.25 ± 1.79
Bio-Filler w/solid	26.04 ± 0.47
